# A Patient-Specific Foot Model for the Estimate of Ankle Joint Forces in Patients with Juvenile Idiopathic Arthritis

**DOI:** 10.1007/s10439-015-1451-z

**Published:** 2015-09-15

**Authors:** Joe A. I. Prinold, Claudia Mazzà, Roberto Di Marco, Iain Hannah, Clara Malattia, Silvia Magni-Manzoni, Maurizio Petrarca, Anna B. Ronchetti, Laura Tanturri de Horatio, E. H. Pieter van Dijkhuizen, Stefan Wesarg, Marco Viceconti, MD-PAEDIGREE Consortium

**Affiliations:** Department of Mechanical Engineering, University of Sheffield, Pam Liversidge Building, Sheffield, S13JD UK; INSIGNEO Institute for in silico Medicine, University of Sheffield, Sheffield, UK; Department of Mechanical and Aerospace Engineering, Sapienza University of Rome, Rome, Italy; Pediatria II ‐ Reumatologia, Istituto Giannina Gaslini, Genoa, Italy; Pediatric Rheumatology Unit, IRCCS Ospedale Pediatrico Bambino Gesù, Rome, Italy; Movement Analysis and Robotics Laboratory (MARLab), Neurorehabilitation Units, IRCCS Ospedale Pediatrico Bambino Gesù, Passoscuro, Rome, Italy; UOC Medicina Fisica e Riabilitazione, IRCCS Istituto Giannina Gaslini, Genoa, Italy; Department of Imaging, IRCCS Ospedale Pediatrico Bambino Gesù, Rome, Italy; Paediatric immunology, University Medical Centre Utrecht Wilhelmina Children’s Hospital, Utrecht, The Netherlands; Visual Healthcare Technologies, Fraunhofer IGD, Darmstadt, Germany

**Keywords:** Musculoskeletal, Sensitivity, Lower-limb, Foot, OpenSim, NMS-Builder

## Abstract

Juvenile idiopathic arthritis (JIA) is the leading cause of childhood disability from a musculoskeletal disorder. It generally affects large joints such as the knee and the ankle, often causing structural damage. Different factors contribute to the damage onset, including altered joint loading and other mechanical factors, associated with pain and inflammation. The prediction of patients’ joint loading can hence be a valuable tool in understanding the disease mechanisms involved in structural damage progression. A number of lower-limb musculoskeletal models have been proposed to analyse the hip and knee joints, but juvenile models of the foot are still lacking. This paper presents a modelling pipeline that allows the creation of juvenile patient-specific models starting from lower limb kinematics and foot and ankle MRI data. This pipeline has been applied to data from three children with JIA and the importance of patient-specific parameters and modelling assumptions has been tested in a sensitivity analysis focused on the variation of the joint reaction forces. This analysis highlighted the criticality of patient-specific definition of the ankle joint axes and location of the Achilles tendon insertions. Patient-specific detection of the Tibialis Anterior, Tibialis Posterior, and Peroneus Longus origins and insertions were also shown to be important.

**Electronic supplementary material:**

The online version of this article (doi:10.1007/s10439-015-1451-z) contains supplementary material, which is available to authorized users.

## Introduction

Juvenile idiopathic arthritis (JIA) is the leading cause of childhood disability from a musculoskeletal disorder. It is a complex autoimmune disease, whose aetiology is still unknown, and it affects between 0.16 and 4 children per 1000.^31^ Any joint can be affected with prevalence of large joints such as the knee and the ankle.^31^ It is characterised by a chronic inflammatory process primarily targeting the synovial membrane; in the most severe cases persistence of inflammation may lead to an increased risk of osteocartilagineous damage and consequent physical functional disability. For example, in a long-term follow-up study it was found that 42.9% of patients with long‐standing JIA (disease duration > 28 years) had a severe disability.^27^

Different factors can contribute to the onset of structural damage to the joint. Recently it has been hypothesized that altered joint loading and other mechanical factors, due to pain and inflammatory processes, may influence the disease progression.^23^ Musculoskeletal modelling can predict patients’ joint loading (joint reaction forces; JRF) and is thus a valuable tool in understanding the disease mechanisms involved in structural damage progression. Previously, musculoskeletal models have been used to design joint replacements,^15^,^28^ analyse diseases,^20^ and develop multi-scale models that have been applied to disease.^42^

Standard modelling practice involves scaling a generic adult model onto each patient, based on palpation of bony landmarks.^3^,^14^,^17^,^25^,^26^,^33^ However, loading conditions like the ankle JRF are sensitive to muscle moment arms^1^ and patient-specific moment arms can differ significantly from a scaled generic model.^2^,^16^ The accuracy of scaled generic musculoskeletal models is being increasingly questioned for: estimating a subject’s musculoskeletal geometry,^8^,^11^ calculating joint kinematics and joint centres^12^,^24^,^34^ and predicting moment arms and muscle-tendon lengths.^1^,^8^,^35^

Patient-specific models and modelling techniques are gaining greater attention and credence.^4^–^6^,^22^,^29^,^35^,^40^ However, lower-limb musculoskeletal models have tended to focus on the analysis of the hip and knee joints.^3^,^9^,^14^,^17^,^25^,^40^ Recent work has presented a generic adult model with an increased level of detail in the foot, including the intrinsic muscles and ligaments of the foot and ankle.^33^ Despite this being a step forward, the model is still generic and has not been used for the creation of patient-specific models.

The aim of this paper is therefore to develop a modelling pipeline that allows the creation of juvenile patient-specific models that include high levels of detail at the foot. This pipeline will be based on data measured with standard techniques such as clinical gait analysis (CGA) and MRI scans of the ankle and foot. The importance of patient-specific parameters and modelling assumptions will be tested in a sensitivity analysis, which aims to highlight where the focus of patient-specificity in models should be targeted in future modelling. The described pipeline will also make it possible to quantify, in future work, the differences that arise between generic and patient-specific models.

## Materials and Methods

### Patients

Three JIA patients (Table 1) participated in an ongoing prospective, longitudinal study, performed at the Ospedale Pediatrico Bambino Gesù (Rome, Italy) and the Istituto Giannina Gaslini (Genoa, Italy). Written informed consent was obtained from all patients and/or their parents. The study was approved by the local medical ethics committees of the participating centres and conducted according to good clinical practice guidelines and the declaration of Helsinki.

**Table 1:** Patient data for the three JIA patients

	Patient 1	Patient 2	Patient 3
Age (years)	15.9	12.9	9.5
Height (m)	1.45	1.53	1.37
Mass (kg)	50.0	64.2	40.6
BMI (kg/m^2^)	23.8	27.2	21.5
Gait Laboratory (code corresponding to the laboratory)	L1	L2	L1

### Data Collection and Pre-processing

Gait analysis data were collected across two laboratories (L1 and L2; Table 1) using an 8-camera stereophotogrammetric system (Vicon, MX, 200 Hz) and two force plates (AMTI, OR6, 1 kHz) in L2, and a 6-camera system (BTS, Smart DX, 100 Hz) with two force plates (Kistler, 1 kHz) in L1. The marker set included all of the markers in the modified Oxford Foot Model (mOFM)^37^ and the Plug-in Gait protocol.^43^ Five gait trials were performed asking the children to walk at their self-selected speed, with a subset of three trials randomly chosen for the analysis.

MRI scans of the distal tibia and complete foot were completed for each patient. The first sequence was a multi-slice multi-echo 3D Gradient Echo (mFFE) scan with water-only selection. These were sagittal plane scans with 1 mm slice thickness, −0.5 inter-slice gap and 0.5 mm in-plane resolution. The bone geometries were segmented from the resulting DICOM data. The second scan was a 3D short T1 inversion time inversion recovery fast field echo scan. These were sagittal plane scans with 2 mm slice thickness, −1 mm inter-slice gap and 0.6 mm in-plane resolution. The muscle paths were determined from the resulting DICOM data.

### Model Components

A generic musculoskeletal model of the lower limb was constructed—to act as a template upon which the patient-specific model could be built. The lower limb and intrinsic foot muscles and ligaments have been defined using the Arnold *et al*.^3^ and Saraswat *et al*.^33^ geometries, respectively. These muscles were defined on the bony geometry of the Arnold *et al*.^3^ model. The foot was also split into three segments according to the modifed Oxford Model (mOFM).^37^ This gave a model with seventeen degrees of freedom (DoF)—six at the pelvis, three at the hip, one at the knee, three at the ankle, three between the hindfoot and forefoot (the metatarsals), and one between the forefoot and the toes. The muscles were represented by fifty-four muscle paths on each limb describing thirty-nine distinct muscles, sixteen of which cross the ankle or the internal joints of the foot. Despite seven foot ligaments being included in this generic model—six crossing the ankle and the seventh being the plantar fascia—they were not included in the simulations performed within this study. This choice was made because the available knowledge about their mechanical properties, which might influence the model output, is still far from being conclusive.^32^ The muscle properties were taken from the Arnold *et al*.^3^ model, as the more complete model. The properties and insertion points of the *brevis* heads of the extensor and flexor muscles were taken from the Saraswat *et al*.^33^ model, since these were not in the original Arnold model. The muscles were first scaled to be in-line with the muscles of the Arnold model crossing the ankle. This model only acted as a first estimate for the patient-specific foot model and is therefore not described exhaustively.

### Patient-Specific Modelling Pipeline

**Figure 1:**
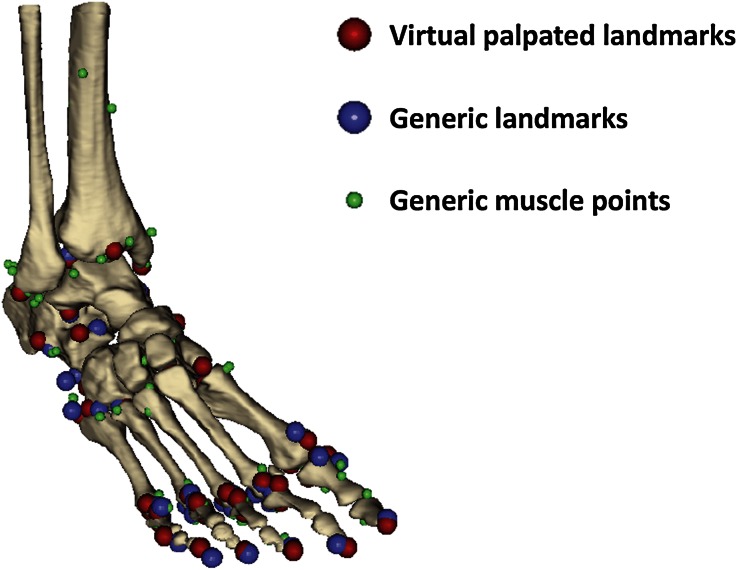
Illustration of the result of the virtual palpation, as obtained from NMS-Builder.

The segmented DICOM data gave the patient-specific foot and distal tibia geometry. Landmarks were identified on the bone geometries (virtual palpation) and used to register the generic model’s muscle attachment (i.e., origin and insertion) and via points (i.e., the points needed to account for the constraints encountered by the muscle path between origin and insertion) for the hindfoot (ten landmarks), talus (six landmarks), metatarsal (fifteen), and toe (seventeen) segments onto matching virtually palpated landmarks on the patient-specific foot geometry (Fig. 1). The markers were distributed all over the segments surfaces and included those proposed by van Sint Jan^41^ as being the simplest to identify (full list in Supplementary 1). The registered muscle attachment and via points acted as a first educated estimate of the patient-specific muscle paths. These muscle points were then adjusted to fit the muscle paths in the patient’s MRI data.

The joint coordinate systems and joint centres of the ankle and foot joints were defined according to the mOFM,^37^*via* virtual palpation of landmarks in the patient-specific geometry. However, a cylinder was fitted to the talar dome to define the ankle joint centre (centre of the cylinder) and the flexion/extension axis of the ankle—with the lateral and medial axes of the cylinder replacing the lateral and medial malleoli used in the mOFM.^37^

Not having the MRI available for the entire lower limb, the proximal segments of the lower limb generic model (pelvis, femur, shank) were scaled based on the markers in the mean static gait analysis trial. The pelvis was scaled^2^ based on: (a) Distance from the mid-point of the anterior superior iliac spines (ASISs) to the mid-point of the posterior superior iliac spines (PSISs; depth); (b) distance from the mean point from the anterior superior iliac spines and the posterior superior iliac spines to the mid-point of the right and left femoral greater trochanters (height); and (c) distance from the mid-point of the right anterior and posterior superior iliac spines to the mid-point of the left anterior and posterior superior iliac spines (width). The femur and shank were scaled based on the distance from the greater trochanter to the mid-point of the femoral epicondyles and the distance from the mid-point of the femoral epicondyles to the mid-point of the malleoli. The scaled generic shank was then registered onto virtually palpated landmarks on the patient’s distal shank geometry (Table in Supplementary 1). The coordinate frames and wrapping objects of the generic model^3^ were scaled according to the same criteria. This included the knee coordinate system used by Arnold *et al*.^3^ and the equations reported by Walker *et al*.^44^ for the derived translations and rotations (anterior/posterior and medial/lateral translation and internal/external and varus/valgus rotation). Thus, a complete lower limb model was created with a patient-specific foot and ankle.

Radio-opaque markers were placed on the patients’ skin, replicating the position of selected gait analysis markers (lateral malleolus, medial malleolus, head of the fifth metatarsal, between second and third metatarsal heads, head of the first metatarsal and base of the hallux) previously marked with an ink pen, so that they were visible in the MRI scan of the foot. The position of each marker in a static (standing) gait analysis trial was averaged in time. A rigid registration was then performed from the averaged static gait analysis markers of the foot (including the malleoli markers) to the gait analysis markers included in the MRI scan of the foot. Similarly, a rigid transformation was performed from the static gait markers of the leg to the malleoli markers in the MRI and the two femoral condyles (virtually palpated in the scaled generic geometry). Once this registration was performed, the femur markers were rotated around the knee flexion/extension until a minimum distance was found between the virtually palpated greater trochanter and the greater trochanter gait analysis marker. Finally, a rigid transformation was performed from the static gait markers of the pelvis to the ASIS and PSIS markers in the MRI to the corresponding points virtually palpated in the scaled generic geometry. All of these procedures were performed in NMS-Builder (simtk.org/home/vphop),^39^ where the registration technique implemented follows the method proposed by Horn.^18^

The whole process of creating a patient-specific model is estimated to take 8–10 h for an experienced operator.

### Model Simulation

The gait analysis markers were used to drive the model’s motion, *via* OpenSim’s Inverse Kinematics routine. Static Optimization (minimising the sum of the squared muscle activations) and Joint Reaction Analysis were used to compute the ankle joint forces.^13^ In the Static Optimisation the fore/hindfoot and the internal/external ankle rotations were locked, and thus the associated moments were not required to reach static equilibrium. The fore/hindfoot was not solved in the static optimisation because the ground reaction force data did not allow a distribution of the load across the three foot-segments. The internal/external rotation of the ankle was not solved because it is assumed that the bony constraints of the ankle complex^19^ and the ligaments of the ankle^10^,^38^ will satisfy the inverse dynamics moments around this axis. When only the active element of the model, i.e., the muscles, are left to satisfy the ankle internal/external rotation moments a solution is not found.

### Sensitivity Analysis

Sensitivity to segment idealisation was tested by analysing two cases of possible ground reaction force (GRF) application. In the one-segment assumption (1SEG) the GRF was applied to the hindfoot segment throughout the trial, thus underestimating the loading of the toe segment. In the two-segment assumption (2SEG) the GRF was applied to the hindfoot segment until the centre of pressure crossed the metatarsophalangeal joint’s flexion/extension axis. At this point the GRF was applied entirely to the toe segment, thus overestimating the toe loading.

**Figure 2:**
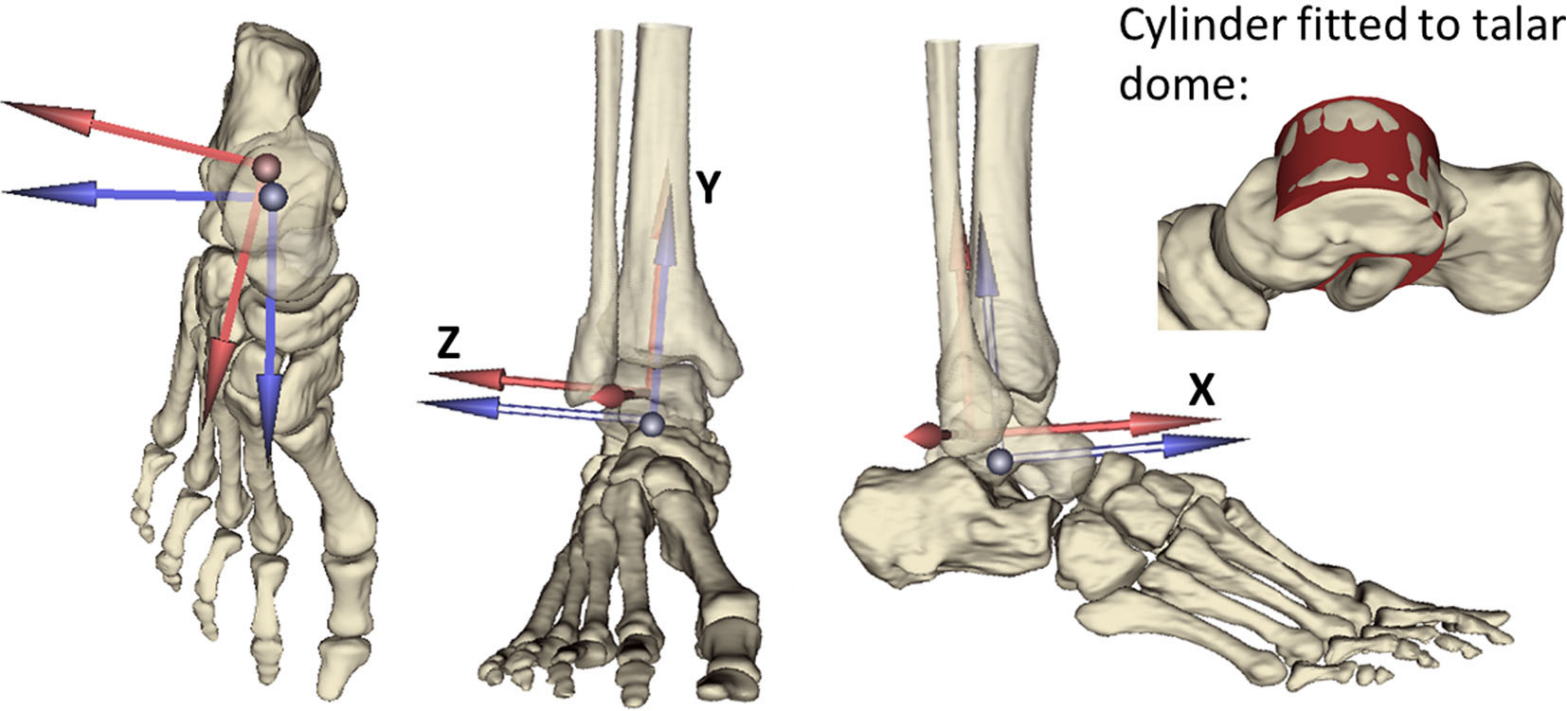
Illustration of the parent coordinate frame of the ankle constructed from MRI (blue) and CGA (red) in a representative patient (Patient 1). Superior, anterior, and lateral views of the foot and distal shank. Also includes an example of the cylinder fitted to the talar dome.

The model’s sensitivity to the ankle joint’s axes definition was tested by analysing two cases (Fig. 2): in the MRI-based assumption (MR_axes) the ankle coordinate frame was defined based on a cylinder fitted to the talar dome, as described in the “Patient-Specific Modelling Pipeline” section. In the CGA-based assumption (CGA_axes) the coordinate frame was defined based on the CGA markers, registered onto the model’s geometry, according to the mOFM.^37^ The mOFM defines the shank, and therefore ankle parent, frame as: a vertical axis from mid-point between the medial and lateral malleolus to the knee joint centre, the anterior axis perpendicular to the plane defined by vertical axis and the vector from the medial to lateral malleolus, and the transverse axis mutually perpendicular.

Sensitivity to muscle attachment points was tested by perturbing each of the points representing the muscles that cross the ankle by 5 mm in the hindfoot coordinate frame. This value was chosen as a reasonable value for human error in virtual palpation of an MRI dataset. A consistent value across muscle points also allows a comparison of the muscles’ relative sensitivities. Only points that were immediately to either side of the ankle joint were used. Perturbations were applied to the Achilles tendon insertion and the following muscles: Tibialis Anterior, Tibialis Posterior, Peroneus Longus, Peroneus Tertius, Peroneus Brevis, Flexor Hallucis Longus, Flexor Digitorum Longus, Extensor Hallucis Longus, and Extensor Digitorum Longus.

## Results

**Figure 3:**
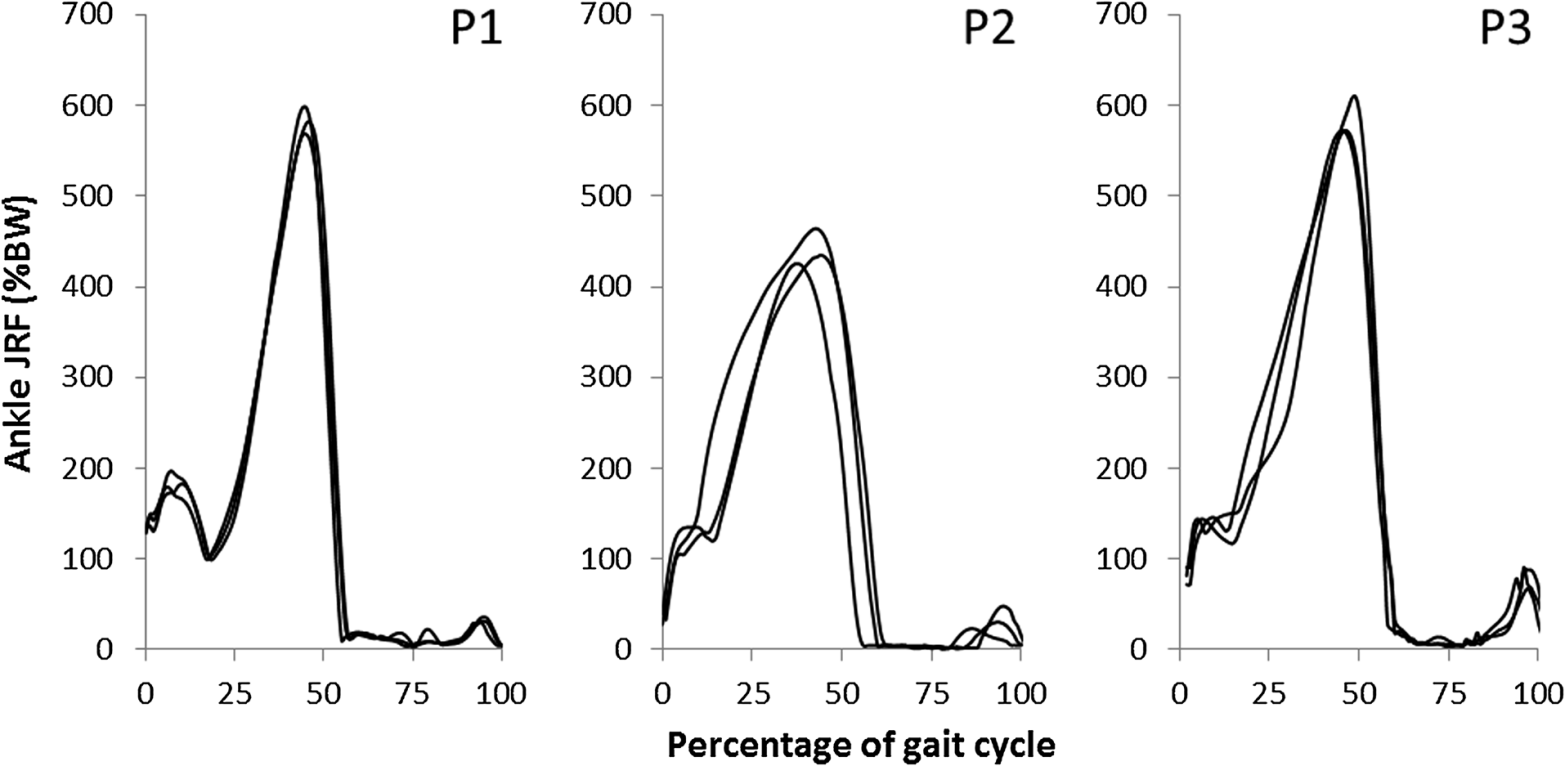
Resultant ankle joint reaction forces (shown as percentage body weight; %BW) in three patients (P1, P2, P3) across three gait trials (all shown in black).

**Figure 4:**
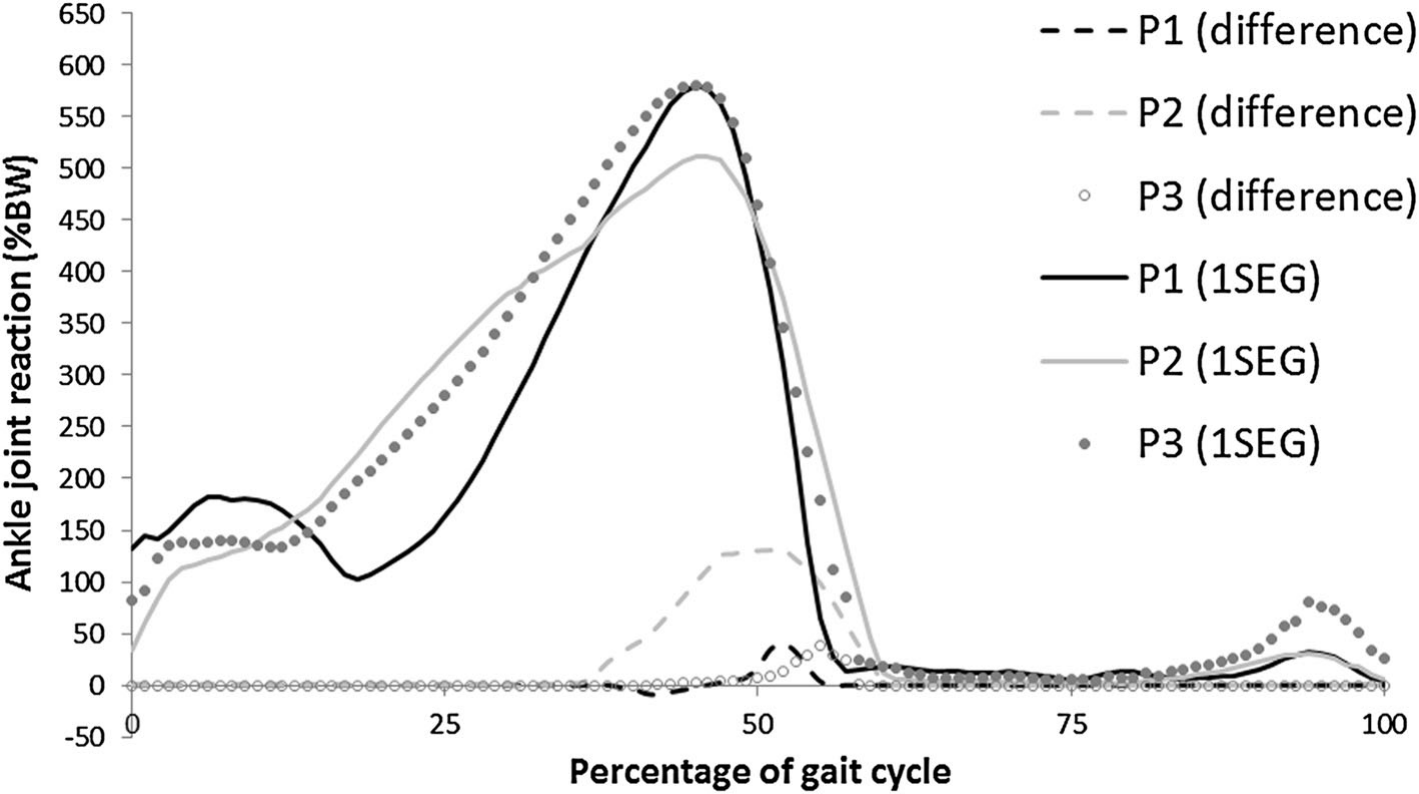
Mean ankle joint reaction force (in percentage body weight; %BW) with the difference between the one (1SEG) and two (2SEG) segment models (2SEG–1SEG).

The ankle joint reaction forces are consistent between each patient’s trials (Fig. 3). The peak ankle joint reaction forces are just above six times body weight in two patients and about four and a half times body weight in the other. Interestingly, qualitative inspection of the two labs’ kinematics and GRF patterns did not show differences that would immediately justify the differences in the JRFs computed for the three patients. The intra-patient similarities justify the presentation of each patient’s mean joint reaction force (Fig. 4).

The effect of the 1SEG vs. the 2SEG assumption appears to depend on the patient being considered; here the JRF computed for Patient 2 (P2) is more affected by the change in segment assumption (Fig. 4). The peak difference in this case is 1.3 times body weight at a peak joint reaction force of 5.1 times body weight (i.e., 25.5% of peak). This is a large effect. The other patients show a small effect—less than 0.35 times body weight in both cases (approximately 6.9% of peak).

**Figure 5:**
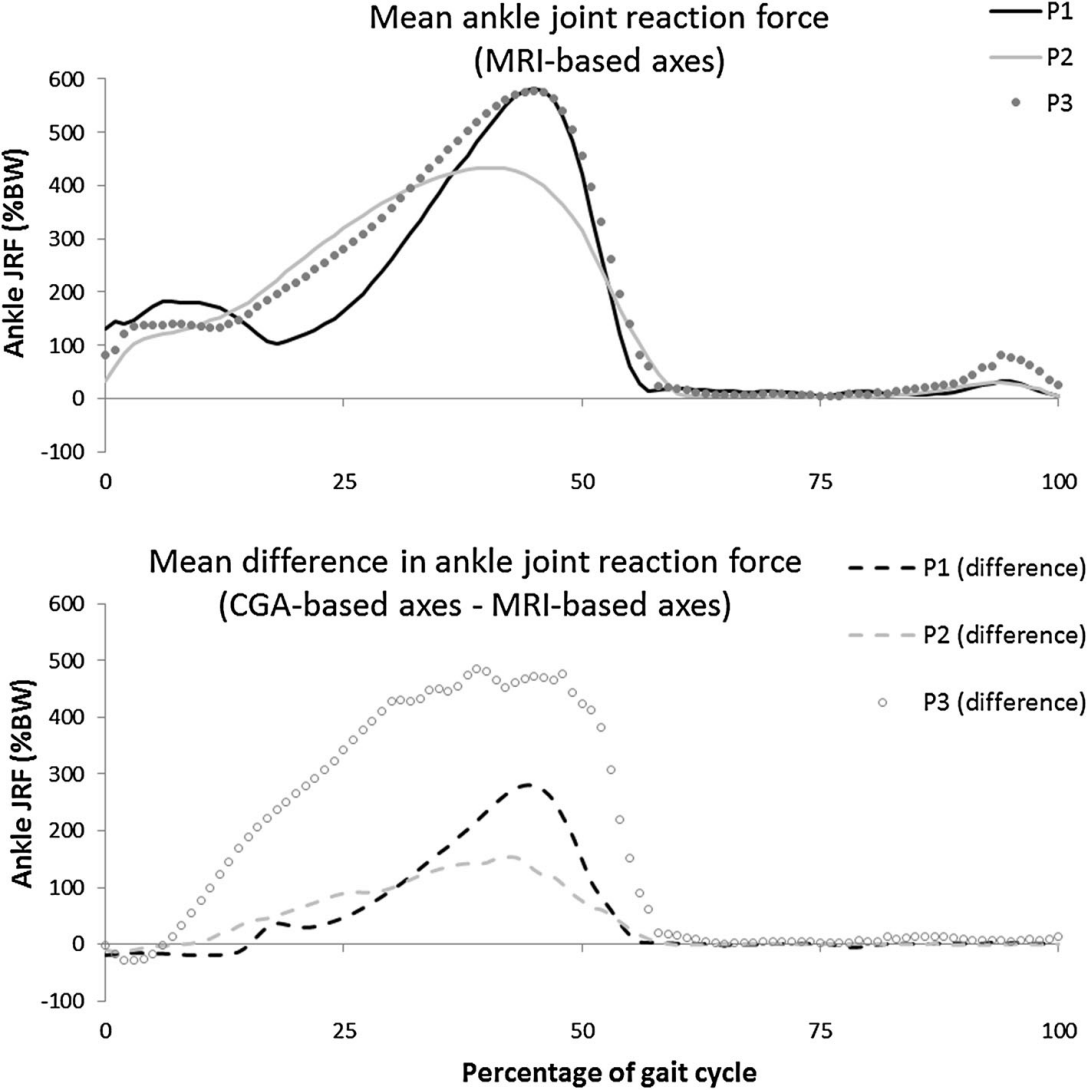
Effect of different ankle joint coordinate frame definitions on the ankle joint reaction force expressed as difference between the two modalities.

**Figure 6:**
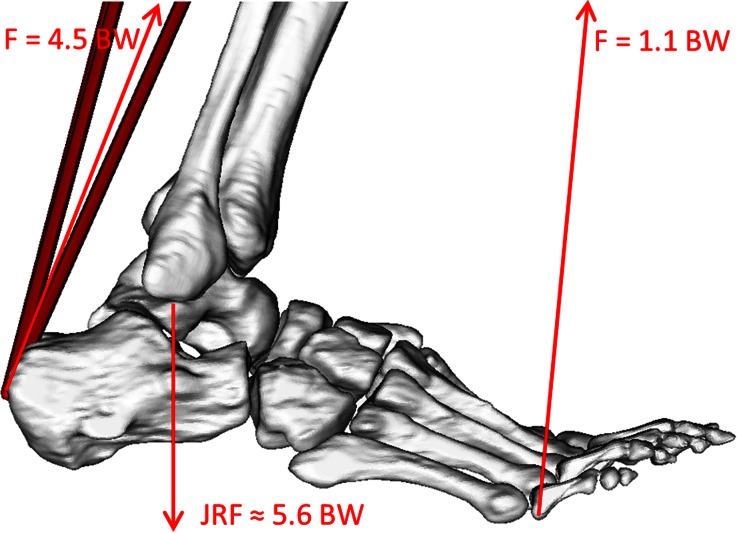
Free-body diagram showing a highly simplified representation of the predicted ankle joint reaction force at the second peak of the GRF in gait. The Achilles force of 4.5 time body weight (4.5 BW) is computed as the force required to balance the moment produced by the ground reaction force. The values and image are taken from Patient 1 represented in OpenSim.

The effect of different ankle joint coordinate frame definitions is very large (Fig. 5). Again, the effect is patient-dependent, but in the smallest case the change is 1.5 times body weight at a peak ankle joint reaction force of 4.3 times body weight. This large effect relates to the inherent sensitivity of the foot geometry to changes in loading position (relative to the joint centre; Fig. 6), as well as the increase in pronation/supination moments required due to the shifted coordinate frame (Fig. 2).

Model-predicted ankle joint reaction force sensitivity to perturbation of muscle paths appears to be high (Table 2). This is particularly true in the case of the Achilles tendon, where a 5 mm movement in the insertion point gives up to a mean change of 7.2%, and up to a maximum change of 13.4% in the ankle joint reaction force magnitude (Tables 2 and 3). Ankle joint reaction force also appears to have sensitivity to the Tibialis Posterior muscle via points in all patient models. Some patient models also show some sensitivity to the Peroneous Longus via points and the Tibialis Anterior via points and insertion. The models showed negligible sensitivity to other muscles crossing the ankle, all of which caused mean changes in the ankle JRF of less than 0.5% across stance.

**Table 2:** Mean percentage change in ankle joint reaction force

Mean values have been computed across the stance phase of gait and across the three trials—original muscle position value subtracted from perturbed muscle position value. Muscles are included that have a mean percentage change of greater than or equal to 0.5% in at least one perturbation in one patient. The colour scale is based on the absolute values and ranges from 7.2 (the maximum value with the highest level of shading) to 0 (with a white background colour). Via points are indicated as “via1, via2, and via3”, whereas the insertion points are indicated as “I”

**Table 3:** Maximum value of percentage change in ankle joint reaction force

Values (mean over three gait trials) have been calculated in the stance phase of gait—original muscle position value subtracted from perturbed muscle position value. Muscles are included that have a mean percentage change of greater than or equal to 0.5% in at least one perturbation in one patient. The colour scale is based on the absolute values and ranges from 13.4 (the maximum value with the highest level of shading) to 0 (with a white background colour). Via points are indicated as “via1, via2, and via3”, whereas the insertion points are indicated as “I”

## Discussion

A pipeline has been described that allows the creation of a lower limb model with a patient-specific foot. The results of the three created models have been presented (Fig. 3) and the magnitude and pattern of the ankle joint reaction forces is similar to other literature models of adults (peaks of 3.9–6.1 BW in literature compared to 4.2–6.1 BW in Fig. 3).^30^,^39^ The order of magnitude of the peak ankle joint reaction forces are according to expectations, based on a free-body diagram analysis of the two peaks in the GRF during gait (Fig. 6).

Sensitivity to the number of segments in the model appears to be dependent on the particular patient (Fig. 4). With the 1SEG modelling assumption, the ground reaction force was applied to the hindfoot segment throughout the trial, thus underestimating the loading of the toe segment. In the 2SEG modelling assumption the GRF was applied entirely to the toe segment, thus overestimating the toe loading—since the metatarsal heads share some of the load during the push-off phase.^21^ Thus, the difference between the 2SEG and 1SEG assumptions will be overestimated. In addition, patients with morphological characteristics, for example flat floot, might have a more even force distribution, making the assumption less realistic. Measurement of pressure sensor contours would allow more detailed analysis of segment and GRF assumptions. Further studies, including the measurement of a pressure sensor contours, might allow to obtain a more accurate distribution of the GRF. Nonetheless, it appears that high sensitivity can exist to the number of segments in a foot model.^45^ An earlier transfer of the load onto the toe segment is likely to make the assumption about the number of segments more important. Given the observed patient dependency of the sensitivity to the number of segments, and the present lack of tools to accurately measure the GRF distribution within a clinical setting, the adoption of a 1SEG modelling assumption should currently be considered as the preferred option.

The effect of using an ankle joint axis based on the dome of the talus—which is the articulating surface of the ankle joint—has been shown to be large (Fig. 5). There is evidence that perturbations in the markers have a small effect on muscle activation patterns during gait.^24^ However, these perturbations were very small relative to the re-definition of the coordinate frame that is used here.

It should be noted that reserve actuators^13^ were required around the *x*-axis of the ankle (Fig. 2) in order to give the model enough strength to solve the static optimisation when a CGA-based ankle coordinate frame was used. It is likely, therefore, that the effect of these coordinate frame definitions is actually under-estimated here. Previous work has assumed that the flexion/extension axis lies along the malleolar axis.^3^,^25^ The talar dome surface can be modelled as a skewed truncated conic saddle shape with a laterally oriented apex,^36^ rather than medially as postulated by other authors.^19^ However, the practical challenges of fitting this shape to the surface outweighed the advantages in this pipeline.

Correct placement of muscle paths has been shown to be large and significant in the determination of joint forces and muscle activation patterns in the more proximal joints of the lower-limb.^7^,^8^,^39^ It has been shown (Tables 2 and 3) that the placement of muscle paths has a large effect on the ankle joint reaction force as well. Therefore, the adjustment of muscle paths according to the patient’s geometry will provide a very significant improvement from the current modelling practice of scaling a generic adult’s geometry to create a model.

The Achilles tendon insertion has been shown to have a very large effect on the ankle joint reaction force—causing changes up to 13.4% for a movement of only 5 mm. This is expected given the key role that the tendon has in providing the force during the push-off phase of gait (Fig. 6). Other literature has also shown a large effect of the Achilles tendon, relative to other muscle points, on the activation of the muscles of the lower limb.^8^

Model predictions of ankle JRF also appear to be sensitive to the anterior/posterior and medial/lateral positioning of the Tibialis Posterior muscle—with mean changes up to 5.1% and maximal changes of up to 8.2% (Tables 2 and 3). Even if this variable has not been calculated to confirm this hypothesis, this phenomenon is likely to be related to the large inversion moment that this muscle typically provides at the ankle. This muscle also has a significant action in plantar flexing the foot, just as the Achilles tendon does. Sensitivity to the Tibialis Anterior (via point and insertion) and the Peroneus Longus (via point) is generally only different over a small period of time. This leads to large maximal changes (Table 3) and relatively small mean changes over the gait cycle (Table 2). The magnitude of these changes is generally smaller than those seen for the Tibialis Posterior, and always smaller than those seen for the Achilles tendon insertion.

The other muscles that were analysed (Peroneus Tertius, Peroneus Brevis, Flexor Hallucis Longus, Flexor Digitorum Longus, Extensor Hallucis Longus, Extensor Digitorum Longus) had a negligible effect on the ankle JRF estimate. The mean percentage change in ankle JRF was less than or equal to 0.5% for perturbation of these muscles.

It was assumed in the registration of the gait analysis markers with the patient-specific geometry, that the foot was rigidly transformed between the MRI scan and the standing gait analysis trial. This is a limitation that may have led to some registration error. However, given the limited number of markers available in the MRI scan, this assumption was necessary in order to have an estimate of the hindfoot gait markers relative to the segmented geometry. The malleoli can also be included since it is assumed that the primary rotation of the foot relative to the shank is around the flexion/extension axis, approximated by the malleoli.

Results of this study are limited by the fact that data from only three subjects have been used and that they came from two different gait laboratories. Nevertheless, results obtained from the sensitivity analysis showed no clear relationship to the lab where the data were acquired. Pressure data at the foot/ground interface would have allowed a more detailed analysis of the segment assumptions and possibly a relaxation of the assumption that the fore/hindfoot should not be optimised. A comparison with a generic model has not been performed; although given the high sensitivity to muscle positions and joint coordinate systems at the ankle, there are expected to be large differences. The described pipeline allows for this comparison to be performed, allowing future work to quantify the precise value of using a patient-specific model versus a generic model.

In conclusion, this study showed that extreme care should be paid to the definition of the ankle joint axes when aiming at estimating ankle joint forces. Furthermore, given the very high model sensitivity to the Achilles tendon insertion, this point should be defined as accurately as possible. Care should also be paid to the Tibialis Anterior, Tibialis Posterior, and Peroneus Longus.

## Electronic Supplementary Material

Supplementary material 1 (PDF 52 kb)
