# Supplementary material for: A Patient-Specific Foot Model for the Estimate of Ankle Joint Forces in Patients with Juvenile Idiopathic Arthritis
Source: Ann Biomed Eng. 2015 Sep 15;44:247–57. doi: 10.1007/s10439-015-1451-z (PMC4690839; doi:10.1007/s10439-015-1451-z)
Supplement: Supplementary file 1 — Supplementary material 1 (PDF 52 kb) [file 10439_2015_1451_MOESM1_ESM.pdf]

Landmarks for the registration of the generic model onto the patient-specific model

Table 1: Landmarks for the pelvis and the femur.

Pelvis	Femur
Right ASIS	Superior and posterior point on greater trochanter
Left ASIS	Centre of femoral head (sphere fit)
Right PSIS	Apotheosis of lesser trochanter
Left PSIS	Centre of the greater trochanter
Centre of the right femoral head (sphere fit)	Lateral and medial epicondyle apex
Centre of the left femoral head (sphere fit)	Most distal point of medial and lateral condyle
	Anterolateral and Anteromedial ridge of the patellar surface groove
	Cylinder with centre (cent_cylinder) at mid-point of FCH and avg(FMC & FLC)
	Height 0.02 and positioned to encompass femoral shaft
	Superior and inferior central point on cent_cylinder

Table 2: Landmarks for the shank and patella.

Tibia	Fibula	Patella
Apotheosis of tibial tuberosity	Distal apex of fibula	Apex of inferior patella
Lateral and medial ridge of tibial plateau	Proximal apex of fibula	Centre of medial and lateral edge
Distal apex of tibia (by medial malleolus)	Cylinder with centre at mid-point of FAL and FAX	Centre of patella anterior face
Posteriorlateral corner of distal tibia	Height 0.02 and positioned to encompass fibula shaft	Centre of superiorposterior ridge of patella
Most anterior portion of centre of distal tibia	Most lateral point on cent_cylinder (relative to the tibia shaft)	
Mid-point of inter-condylar tubercles	Most medial point on cent_cylinder (relative to the tibia shaft)	
Cylinder with centre at mid-point of top and TAM		
Height 0.02 and positioned to encompas tibia shaft		
Point at the centre of cent_cylinder		

Table 3: Landmarks for the foot (used also for MRI registration).

**Hindfoot**	**Talus**	**Metatarsals**	**Toes**
Sustentaculum tali (most medial point)	Centre of articulating surface of the tibio-talar joint	Apex of proximal 5th met	Distal point of each of the distal phalanges and the hallux
Apex of posterior calcaneous	Apex of the inferior lateral process	Superior distal head of each metatarsal bone	Superior, inferior and medial point on proximal hallux head
Navicular (big medial bone) tuberosity (mid-point of medial side)	Apex of the anteriormedial tuberosity	Centre of proximal articular surface of the first four metatarsal bone	Superior and inferior point on proximal 2nd, 3rd and 4th phalanx head
Greater apophysis of calcaneous (most superior anterior point)	Most posterior point on the talus	Inferior distal head of each metatarsal bone	Superior, inferior and lateral point on proximal 5th phalanx head
Peroneal trochlea (prominence opposite STL)	Apex of the superior ridge through the centre of the lateral process		
Most inferior point on calcaneous (on floor)	Inferior posteriormedial corner of the talus		
Posteriorinferior corner (2nd most inferior) of cuboid (lateral bone)			
Anteriorinferior corner of cuboid			
Point on tuberosity at the inferioranterior calcaneous (most inferior and anterior)			
Most anterior point on hindfoot (superior too)			
